# Predictive factors for microvascular recovery after treatments for diabetic retinopathy

**DOI:** 10.1186/s12886-023-02788-6

**Published:** 2023-01-25

**Authors:** Junyeop Lee, Yoon-Jeon Kim, Joo-Yong Lee, Young Hee Yoon, June-Gone Kim

**Affiliations:** 1 Department of Ophthalmology, Asan Medical Center, University of Ulsan, College of Medicine, 88 Olympic-Ro 43-Gil, Songpa-Gu, Seoul, 05505 Korea; 2 Asan Diabetes Center, Asan Medical Center, University of Ulsan, College of Medicine, Seoul, Korea

**Keywords:** Diabetic retinopathy, Microvascular recovery, Angiographic findings

## Abstract

**Background:**

To identify factors associated with microvascular recovery after intravitreal bevacizumab or panretinal photocoagulation (PRP) in diabetic retinopathy (DR).

**Methods:**

We retrospectively reviewed 320 eyes/patients with DR treated with intravitreal bevacizumab and/or PRP. Two consecutive fluorescein angiographies (FAs) of each eye were compared. The number of microaneurysms and the area of capillary non-perfusion were calculated automatically using ImageJ software. Microvascular recovery was defined as a marked reduction in the numbers of microaneurysms (< 20%) or a marked reduction in the area of capillary non-perfusion (< 50%) in 45-degree fields or a complete regression of new vessels in ETDRS 7 standard fields. Baseline FA findings and changes in the ocular and systemic factors were analyzed.

**Results:**

Twenty-eight (8.8%) of the 320 total eyes were found to meet the criteria of microvascular recovery after the treatments. Multivariate analysis revealed the presence of diffuse capillary telangiectasis (*P* = .003) and late disc leaking (*P* = .007) on baseline FA and a reduction of glycated hemoglobin (*P* = .005) during the follow-up period were predictive factors of microvascular recovery after the treatments. Although the microvascular recovery group presented with a significant improvement of BCVA after the treatments, the baseline BCVA could not predict the microvascular recovery after the treatments.

**Conclusions:**

Diffuse capillary telangiectasis or late disc leaking on baseline FA and improved glycemic control positively predicted the microvascular recovery after treatments for DR.

## Background

Diabetic retinopathy (DR) is the most common retinal vascular disease [1]. DR is a chronic and progressive disease that is a significant cause of visual loss in working-age people [1]. Timely and appropriate treatments, including control of glycemia, blood pressure, and lipids, can delay its progression over long periods [1]. Local therapies for DR, including panretinal photocoagulation (PRP) and intravitreal anti-vascular endothelial growth factor (VEGF) injections, exhibit rapid therapeutic effects and even reverse the microangiopathy. About half of proliferative DR (PDR) patients respond to PRP with regression of new vessels (NVs) [2, 3]. Intravitreal anti-VEGF drugs are also effective for reversing microaneurysms and persistent NVs [3, 4]. However, there has been no report about the overall rate of microvascular recovery in the real-world clinic after treatments for diabetic retinopathy.

Fluorescein angiography (FA) is useful for evaluating diabetic eye disease, and it is currently the gold standard for evaluating the retinal vasculature [5]. FA is an invaluable adjunct in diagnosing and managing DR. The earliest and most important angiographic findings of DR in FA is a microaneurysm. The number of microaneurysms is an important prognostic indicator for worsening DR [6, 7]. In addition, their formation rate is inversely associated with the duration of diabetes and is directly correlated with the response to treatments [8]. Optical coherence tomography (OCT) angiography has been introduced and can replace FA with certain aspects [9]. However, OCT angiography (OCTA) has several limitations in evaluating leakage, the angle of imaging, and the reliability of the images. Although the use of wide-field angiography has increased recently, the device is not yet universally available in clinics [10].

Because DR is a progressive and life-long disease, the discrimination of treatable and reversible clinical findings and the prediction of the treatment response and disease recovery are essential for long-term treatments to reduce the risk of visual loss. Several clinical trials have reported an improvement of microvascular abnormalities or DR severity score (DRSS) after treatment with intravitreal ranibizumab or aflibercept injections [11, 12]. However, there has been limited research on the correlation between baseline angiographic findings and microvascular recovery and any associated factors affecting the recovery in DR. Therefore, in this study, we aimed to identify the predictive factors among the baseline systemic, ocular, and angiographic factors associated with microvascular recovery on two consecutive FAs before and after treatments for DR.

## Methods

### Study design

The Institutional Review Board approved this retrospective study at Asan Medical Center (IRB number: S2019-2252). This study adhered to the tenets of the Declaration of Helsinki. The Institutional Review Board approved the off-label use of intravitreal bevacizumab (IVB).

### Patients

We included patients with DR diagnosed by fundoscopy, OCT, and FA who were treated with PRP or IVB injections between March 2012 and March 2018 at Asan Medical Center. The indication for PRP was the presence of severe peripheral ischemia, with or without neovascularization, at level 53 or worse on the ETDRS severity scale [13]. IVB was performed mainly to treat diabetic macular edema (DME) and prevent angiogenic episodes and DME before and after PRP depending on the clinicians. If the DME recurred, repeated IVB injections were performed depending on the clinicians’ treatment protocols. The inclusion criteria included patients who underwent two FAs before and after treatments within the period. The interval between two FAs was not more than two years. The follow-up FA was within one year after the last treatments. The exclusion criteria included patients with type 1 DM, a previous history of focal laser, intraocular or periocular steroid injection, vitrectomy, wide-field angiography, poor image quality, and the presence of other retinal diseases. The study eye was selected according to the inclusion/exclusion criteria. If both eyes met the study inclusion criteria, the eye showing the more advanced ETDRS severity level at the baseline FA was chosen as the study eye.

### Systemic characteristics

We investigated the baseline characteristics when the baseline FA was performed: age, sex, duration of diabetes, hemoglobin A1c (HbA1c), body mass index (BMI), underlying systemic disease including hypertension, and dyslipidemia. To evaluate the changes in systemic parameters after treatment, we assessed the HbA1c measurement performed closest in time to the following FA.

### Ophthalmological examinations

All subjects underwent best-corrected visual acuity (BCVA) measurements with a refraction test, slit-lamp examination, and binocular indirect ophthalmoscopy after pupil dilation with 0.5% tropicamide. These examinations were performed at every follow-up visit. BCVA was converted to logMAR (logarithm of the minimum angle of resolution) units. FA was performed using an retinal angiography system (TRC IX50, Topcon, Japan) to diagnose DR. We obtained the central retinal thickness (CRT) from the OCT images using the Heidelberg Spectralis® OCT system (Heidelberg Engineering, Germany). For the evaluation of ocular characteristics, the duration of DR and the stage of DR categorized by FA, BCVA, intraocular pressure (IOP), and CRT were compared between the baseline and the following FA.

### Interpretation of FA

Two retinal specialists (JL and Y-JK) evaluated whether the baseline FA of each eye showed venous beading, arteriolar narrowing, diffuse capillary telangiectasia, intraretinal microvascular abnormality (IRMA), non-perfusion area, extra-retinal NVs, or late disc leaking. When the reviewers agreed, the angiographic findings were considered positive. When disagreed, the results were deemed to be negative.

### Criteria for microvascular recovery

**Fig. 1:**
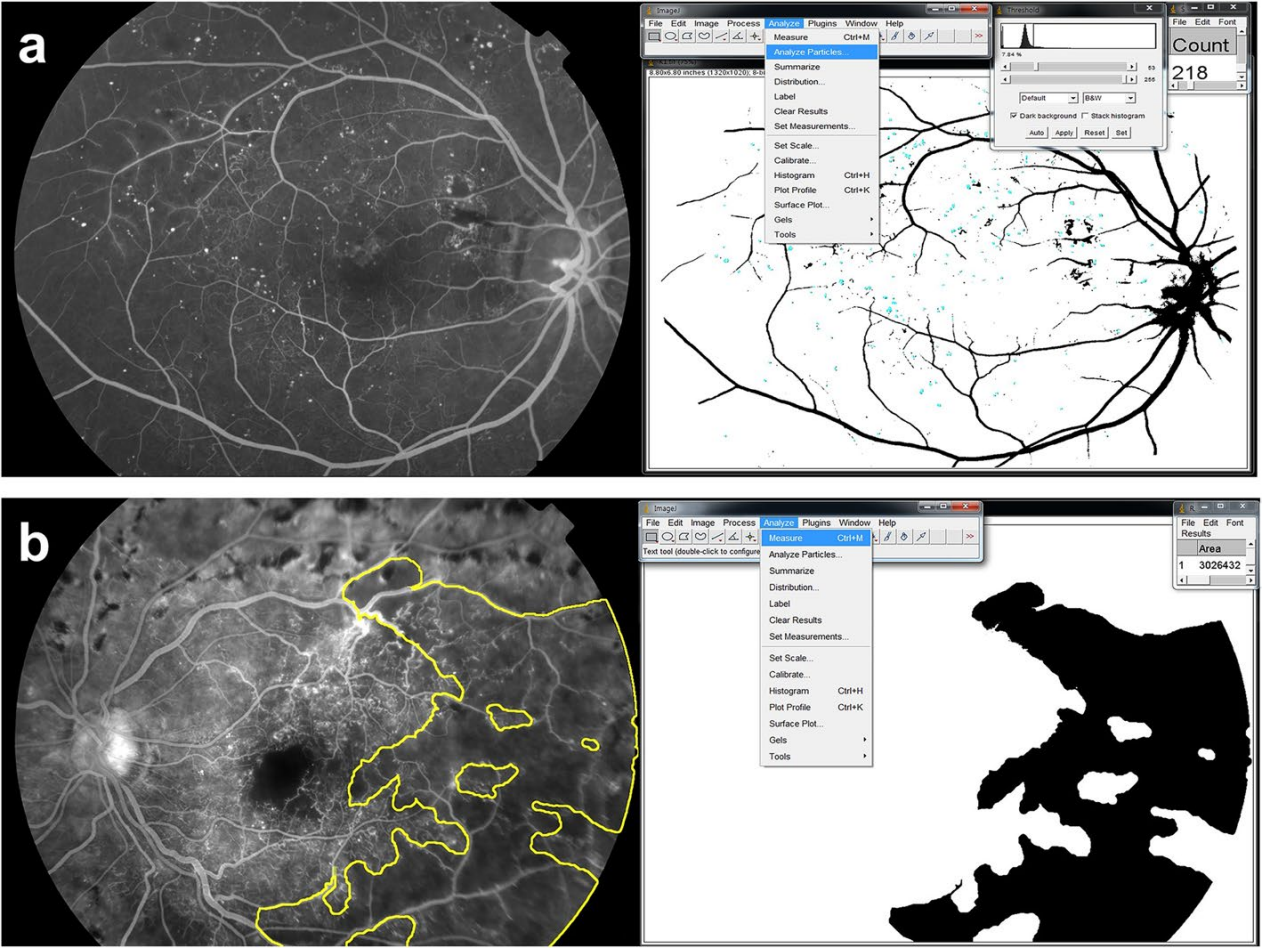
Measurement of the number of microaneurysms (**a**) and non-perfusion area (**b**) in fluorescein angiography using ImageJ software

**Fig. 2:**
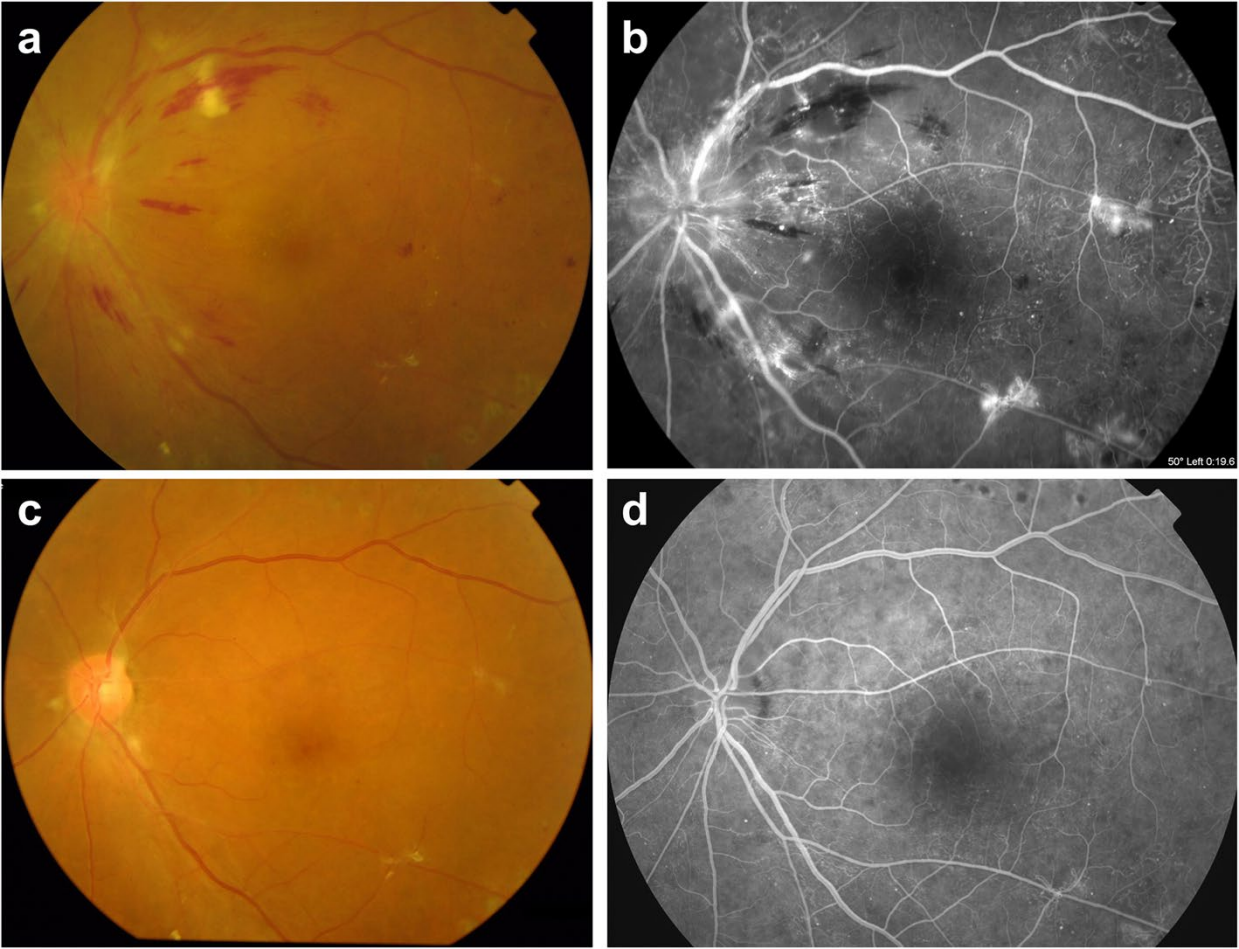
A representative case of microvascular recovery showing complete regression of new vessels (NVs) and a marked reduction in the numbers of microaneurysms after panretinal photocoagulation and intravitreal bevacizumab injection. The presence of NVs and non-perfusion areas (12,945 pixel^2^) and 437 microaneurysms were detected in the baseline fundus photography (**a**) and fluorescein angiography (**b**). After treatments, NV disappeared, the non-perfusion area (7378 pixel^2^) was reduced by 43.0%, and the number of microaneurysms (*n* = 62) was decreased by 85.8% in the following fundus photography (**c**) and fluorescein angiography (**d**)

We selected a mid-phase well-centered FA image from every examination. The numbers of microaneurysms were calculated automatically using ImageJ software (Fig. 1a). After binarization, positive signals ranging between 10 to 100-µm size were counted using the Analyze Particles tool in the operating ImageJ 1.48 software (National Institutes of Health, Bethesda, MD) (Fig. 1a). To measure the non-perfusion area in the 45-degree field posterior pole, a manually demarcated area was analyzed using the Measure tool in the ImageJ software (Fig. 1b). Microvascular recovery was defined as a marked reduction in the numbers of microaneurysms (< 20%), or marked reduction in the area of capillary non-perfusion (< 50%) in 45-degree fields, or complete regression of NVs in ETDRS 7 standard fields. A representative case presenting microvascular recovery is described in Fig. 2.

### Statistical analysis

All statistical analyses were performed using the SPSS 24.0 (IBM Corporation, Armonk, NY, USA) statistical software package. Categorical variables are presented as number and percentage values and were analyzed using the chi-squared test. The Student t-test, Pearson chi-square, and Fisher’s exact test were used to compare the mean values from baseline. Continuous variables presented with the mean, standard deviation (SD), and range were analyzed using Student’s t-test to determine the relationships between the parameters and outcomes. Logistic regression analysis was performed to identify factors associated with the microvascular recovery. A P-value < 0.05 was deemed to be statistically significant.

## Results

### Group division and baseline characteristics

**Fig. 3:**
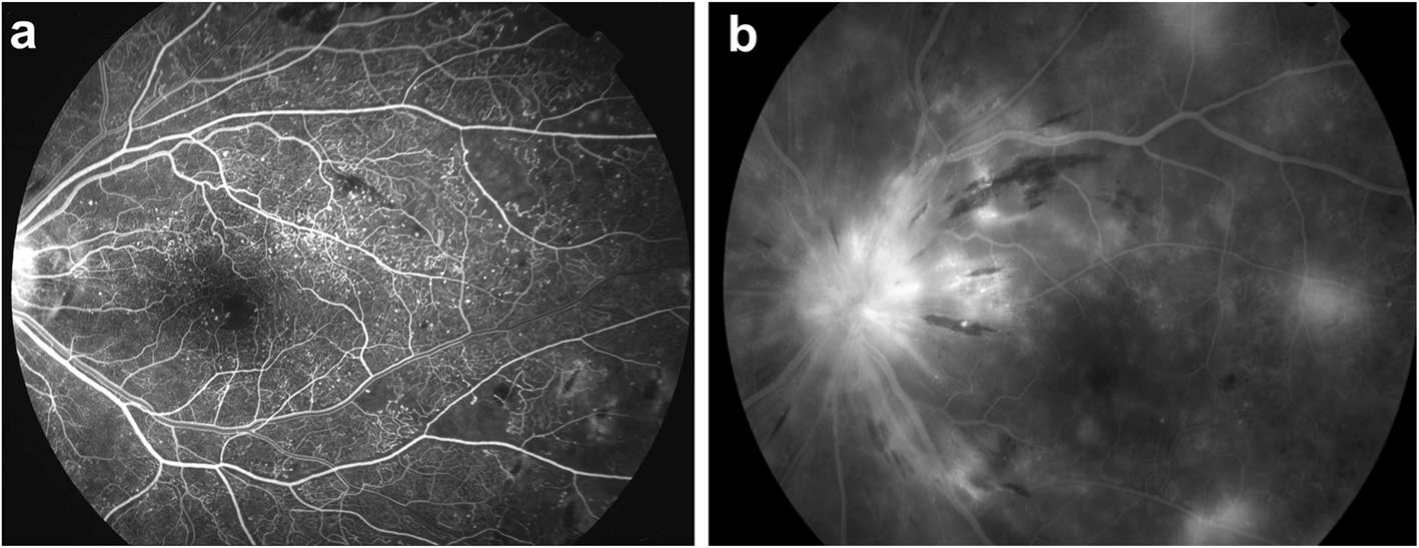
Representative angiographic findings of diffuse capillary telangiectasis (**a**) and late disc leaking (**b**) in the baseline fluorescein angiography

We retrospectively reviewed 320 eyes/patients of DR, which were treated with IVB injections or PRP. Two consecutive fluorescein angiography (FA) of each eye were compared. Microvascular recovery was defined as a reduction in the numbers of microaneurysms, or a reduction in the area of capillary non-perfusion, or complete regression of NVs as described in the Methods section. Among a total of 320 eyes, only 28 eyes (8.8%) presented with microvascular recovery between the two FAs (Table 1). Among 28 eyes, 21 eyes (75.0%) presented reduced number of microaneurysms, 10 eyes (35.7%) showed a reduction in non-perfusion, and eight eyes (28.6%) demonstrated complete regression of NVs. There was no difference in FA intervals between the recovery and the non-recovery group. The duration of diabetes tended to be longer in the non-recovery group than the recovery group, but the difference was not significant. Other baseline systemic factors, including HbA1c, BMI, and systemic diseases, differed between the groups. In addition, ocular factors including the duration of DR, stage of DR, visual acuity, refractive error, IOP, and CRT were not different between the two groups (Table 1). The presence of diffuse capillary telangiectasia and late disc leaking in the baseline FA were more frequently observed in the recovery group (Table 2). Representative FA findings of diffuse capillary telangiectasis and late disc leaking are presented in Fig. 3.

**Table 1:** Baseline characteristics of the patients with diabetic retinopathy according to the microvascular recovery after treatments

	Total	Non-recovery Group	Recovery Group	*P* value
Number of eyes/patients, n (%)	320 (100)	292 (91.2)	28 (8.8)	
Interval between FAs (months)	13.0 ± 4.0	12.9 ± 4.1	13.2 ± 3.8	0.257^a^
** < Systemic characteristics > **				
Age (year)	65.2 ± 11.5	65.4 ± 12.2	64.6 ± 10.9	0.392^a^
Sex (men/women)	211 / 109	193 / 99	18 / 10	0.484^b^
DM duration (year)	9.4 ± 3.5	9.4 ± 3.1	8.6 ± 2.9	0.157^a^
HbA1c (%)	7.54 ± 1.74	7.58 ± 1.95	7.46 ± 1.74	0.464^a^
BMI (kg/m^2^)	27.2 ± 5.6	27.4 ± 5.5	26.7 ± 4.7	0.232^a^
Presence of HTN	125	113	12	0.456^b^
Presence of dyslipidemia	93	84	9	0.514^b^
** < Ocular characteristics > **				
DR duration (year)	2.8 ± 2.5	2.7 ± 2.9	3.2 ± 2.9	0.652^a^
PDR/ severe NPDR	92 / 228	84 / 208	8/ 20	0.357^b^
BCVA (logMAR)	0.36 ± 0.32	0.38 ± 0.31	0.34 ± 0.39	0.246^a^
Refractive error (D)	0.85 ± 0.51	0.86 ± 0.58	0.79 ± 0.52	0.242^a^
IOP (mmHg)	16.7 ± 3.7	16.8 ± 3.8	16.6 ± 2.7	0.328^a^
CRT (μm)	283 ± 111.9	284.9 ± 118.1	277.3 ± 112.4	0.495^a^

*FA* Fluorescein angiography,*HTN* Hypertension, *BCVA* Best-corrected visual acuity, *logMAR* Logarithm of the minimum angle of resolution, *IOP* Intraocular pressure, *CRT* Central retinal thickness

^a^Student *t*-test, ^b^Pearson chi-square or Fisher’s exact test

**Table 2:** Comparisons of baseline angiographic findings between the groups according to microvascular recovery after the treatments

	Non-recovery Group (*n* = 292)	Recovery Group (*n* = 28)	*P* value ^a^
Presence of venous beading	20	1	0.565
Presence of arteriolar narrowing	27	1	0.571
Presence of telangiectasia	17	9	0.003
Presence of IRMA	58	7	0.554
Presence of non-perfusion area	73	6	0.356
Presence of extra-retinal NVs	43	4	0.493
Presence of late disc leaking	6	4	0.003

*IRMA* Intraretinal microvascular abnormality, *NVs* New vessels

^a^Fisher's exact test

### Change of parameters after treatments according to microvascular recovery

In both groups, most eyes/patients were treated with a combination of PRP and IVB, but there was no difference in terms of treatment modalities or the number of IVBs between groups (Table 3). More than 90% of eyes were treated with IVB, with fewer than three injections between the FAs. Intervals between the last treatment and FA were not different between the two groups. During the follow-up period between the two FAs, changes in the systemic ocular parameters were compared. In the recovery group, the average HbA1c decreased after treatment but it increased in the non-recovery group, which was statistically significant (Table 4). Visual acuity was improved in the recovery group but not in the non-recovery group. However, there was no difference between IOP and CRT.

**Table 3:** Comparisons of treatment modalities between the groups according to the microvascular recovery after the treatments

	Non-recovery Group (*n* = 292)	Recovery Group (*n* = 28)	*P* value
** < Treatment modality > **, n (%)			0.632^a^
PRP only	38 (13.0)	4 (14.3)	
IVB only	12 (4.1)	2 (7.1)	
PRP + IVB	242 (82.9)	22 (78.6)	
** < Numbers of IVB > **, n (%)			0.837^a^
0	38 (13.0)	4 (14.3)	
1	79 (27.1)	9 (3.2)	
2	148 (50.7)	14 (50.0)	
> 3	27 (9.2)	1 (0.4)	
** < Intervals between treatment and FA > **			
From baseline FA to initial treatment (mo)	0.1 ± 0.3	0.2 ± 0.7	0.375^b^
From last treatment to follow-up FA (mo)	7.8 ± 3.8	8.7 ± 2.3	0.262^b^

*PRP* Panretinal photocoagulation, *IVB* Intravitreal bevacizumab

^a^Fisher's exact test,^b^Student *t* test

**Table 4:** Changes of systemic and ocular parameters after the treatments according to microvascular recovery after treatments

	Non-recovery Group (*n* = 292)	Recovery Group (*n* = 28)	*P* value ^a^
** < *Systemic parameter* > **			
Δ HbA1c (%)	+ 0.26 ± 0.37	- 0.74 ± 0.44	< 0.001
** < *ocular parameters* > **			
Δ BCVA (logMAR)	+ 0.12 ± 0.11	- 0.05 ± 0.08	< 0.001
Δ IOP (mmHg)	+ 0.6 ± 2.7	+ 0.7 ± 2.1	0.434
Δ CRT (μm)	- 6.1 ± 12.2	- 4.3 ± 3.7	0.465

*BCVA* Best corrected visual acuity, *IOP* Intraocular pressure, *CRT* Central retinal thickness

^a^*t* test

### Factors associated with microvascular recovery after the treatments

Multivariate logistic regression analysis demonstrated that, among several candidate factors, decreased HbA1c and the presence of diffuse capillary telangiectasia and late disc leaking were positive predictors for microvascular recovery after treatment (Table 5). Other factors, including age, DM duration, BCVA, and changes in BCVA were not associated with microvascular recovery after the treatments.

**Table 5:** Multivariate logistic regression analysis to determine the predictive factors of the microvascular recovery after treatments

Factors	*P* value	Odds ratio	95% CI
Age at baseline	0.350	1.34	0.71–2.51
DM duration	0.742	1.19	0.41–3.45
Baseline BCVA (logMAR)	0.127	1.51	0.89–2.57
Δ HbA1c (%)	0.005	0.52	0.40–0.83
Δ BCVA (logMAR)	0.150	0.60	0.30–1.20
Presence of telangiectasia	0.003	9.14	1.92–46.12
Presence of late disc leaking	0.007	7.58	1.91–26.13

*BCVA* Best-corrected visual acuity, *logMAR* Logarithm of the minimum angle of resolution, *CI* Confidence interval

## Discussion

Diabetic retinopathy is a vision-threatening complication of diabetes. Chronic exposure to hyperglycemia impacts the retinal neurovascular unit and its interdependent vascular, neuronal, glial, and immune cells [14]. To avoid retinal neuronal dysfunction in diabetes and the risk of visual loss, preventative or regenerative treatments for the microvascular abnormalities are the primary treatments for DR [14].

In this study, the microvascular recovery rate after IVB or PRP in DR was 8.8%, with no differences among the treatment modalities. The PANORAMA clinical trial reported that 65% to 79% in the aflibercept group showed a 2-step or greater improvement in the DRSS level at 52 weeks [11]. Even in the control group, 15% of eyes showed a 2-step or greater improvement [11]. Based on the photographic features that comprise DRSS grading, it is likely that reductions in retinal hemorrhage do not correlate with actual improvements in retinal vascular perfusion. In the DRCR net, the protocol S study demonstrated that 70% and 65% of eyes treated with PRP and intravitreal ranibizumab, respectively, remained at the PDR and did not improve to the NPDR level after two years of treatment [15]. These results may represent undertreatment, as this study showed that more than 90% of patients were treated with IVB by fewer than three injections between FAs.

Compared to the clinical trials, our study has an advantage in that we assessed the microvascular recovery by analyzing the FAs. We set the strict criteria for microvascular recovery to identify the significant factors predicting the recovery. New microaneurysms are reported to be continually forming while 40% to 80% disappear every year [16]. Although three monthly injections with ranibizumab decreased the total number of microaneurysms by 29%, the untreated control group also showed reduced microaneurysms by 14% for four months [8]. One longitudinal study assessing each microaneurysm's location demonstrated that only 29.4% remained at the exact spot over two years [17]. In the prospective observational study, 76% of microaneurysms were disappeared, but a similar amount of microaneurysms was newly formed during two years [18]. Considering these previous reports, we defined microvascular recovery as less than 20% of microaneurysms compared to the baseline because more than 80% of the patients in our study underwent IVB. To specify the subpopulation that shows the prominent recovery after treatments and to find the associated factors in this study, we had to exclude the possibility of improvement in the number of microaneurysms as a natural course.

For the first time, this study demonstrated that diffuse capillary telangiectasis and late disc leaking in baseline FA were positive predictive factors for microvascular recovery after treatments. Telangiectasis in DR might represent recent up-regulation of VEGF, which could be effectively neutralized through the timely inhibition of VEGF. Although wide-field angiography has been used to assess DR recently, it has limitations to image the diffuse capillary telangiectasis at the posterior pole in detail. Late disc leaking could be considered a finding of diabetic papillopathy, which is reported to regress effectively after IVB [19].

The number of microaneurysms is an essential prognostic indicator for worsening DR [6, 7]. It is necessary to carefully analyze the changes of MAs because they are dynamic: new microaneurysms are continually forming while existing ones disappear [16]. In addition, the formation rate is inversely correlated with the duration of diabetes and is directly associated with the HbA1c values and biomarkers of the response to treatments [8]. The turnover rate of MAs is associated with diabetic retinopathy progression and the development of macular edema [7, 18, 20].In addition, intensive intravitreal ranibizumab decreased the MA turnover [21]. Recently, microvascular changes using OCTA have provided better imaging of microaneurysms and several other biomarkers for DR progression and treatment responses [22, 23]. However, in this study, the microaneurysm are located globally in the 45-degree FA images (Fig. 1a), which could not be detected by conventional 6 × 6 mm OCTA. Additional studies using wide-field OCTA will be promising for validating the reversal or turnover of microaneurysms in DR after treatments.

In areas of capillary nonperfusion in DR, capillary occlusions can promote the progression of retinal disease [24]. These microvascular changes can cause angiogenic complications such as PDR, stimulate the expression of VEGF, and finally cause the breakdown of the blood-retina barrier, which are the major causes of significant DR-related vision loss [24]. There is still controversy whether neutralization of VEGF slows the progression of retinal non-perfusion or not [25, 26]. In this study, a reduction of the non-perfusion area was observed in the microvascular recovery group, although assessed at the posterior pole, which was accompanied by an improvement of other angiographic findings such as disc leaking and telangiectasia (Fig. 2 and Fig. 3).

Systemic glycemic control is important for microvascular recovery, as well as the progression of DR [1]. In this study, baseline HbA1c was not associated with microvascular recovery. However, the reduction of HbA1c during the treatment was a substantial predictive factor for microvascular recovery. Although paradoxical worsening of DR has been associated with rapid improvement of diabetic control [27], the recovery group in this study showed only a -0.74% reduction in HbA1c over 12 months, and baseline glycemic control was not different compared to the previous studies [28]. Therefore, appropriate diabetic control is the only modifiable factor during the treatment in this study.

Baseline BCVA could not predict and is not an associated factor for microvascular recovery. However, visual acuity improvement during the treatment was significant in the recovery group compared to the non-recovery group. Clinical trials including Protocol T support this discordance between the visual acuity and anatomical improvement in DR, showing that moderate correlation potentially derived from retinal atrophy, macular ischemia, or other co-morbidities in eyes with DR [29].

Our study has several limitations. All of the patients were Korean. Selection bias can occur in such a retrospectively designed study. Thus, several confounding factors could not be controlled; for example, treatment protocols, natural course of diseases, the presence of macular edema, or ischemia. Recruiting larger cohorts may be a challenge in a single center and thus warrants multicenter studies on this issue. Wide-field angiography was not used in this study, although the non-perfusion area mainly distrusted the peripheral region [10]. Despite these limitations, our study has advantages in that we included a large number of patients and analyzed the systemic, ocular, and angiographic factors comprehensively in the real-world setting.

## Conclusions

In conclusion, diffuse capillary telangiectasis or late disc leaking on baseline FA and improved glycemic control during the treatments positively predicted microvascular recovery after treatments for DR.

## Data Availability

The data and materials are presented within the manuscript.

## Abbreviations

PRP: Panretinal photocoagulation
DR: Diabetic retinopathy
FA: Fluorescein angiography
VEGF: Vascular endothelial growth factor
PDR: Proliferative diabetic retinopathy
NV: New vessels
OCT: Optical coherence tomography
OCTA: Optical coherence tomography angiography
DRSS: Diabetic retinopathy severity score
IVB: Intravitreal bevacizumab
DME: Diabetic macular edema
HbA1c: Hemoglobin A1c
BMI: Body mass index
BCVA: Best-corrected visual acuity
CRT: Central retinal thickness
IOP: Intraocular pressure
IRMA: Intraretinal microvascular abnormality
SD: Standard deviation
